# Odontogenic Sinusitis: Clinical, Radiologic, and Quality-of-Life Characterization in a Prospective Study

**DOI:** 10.3390/jcm15197336

**Published:** 2026-09-22

**Authors:** Narjesse Kemcha, Helena Rey-Martínez, Pedro Luis Ruiz-Sáenz, Cristina Barona-Dorado, José María Martínez-González, Natalia Martínez-Rodríguez

**Affiliations:** 1 Department of Dental Clinical Specialties, Faculty of Dentistry, Complutense University of Madrid, 28040 Madrid, Spain; nkmcha@ucm.es (N.K.); jmargo@ucm.es (J.M.M.-G.); nataliamartinez@ucm.es (N.M.-R.); 2 Department of Otolaryngology, Central Hospital of the Red Cross of Madrid, 28003 Madrid, Spain; helena.rey@salud.madrid.org; 3 Department of Dentistry, Central Hospital of the Red Cross of Madrid, 28003 Madrid, Spain; drruizsaenz@gmail.com; 4 Surgical and Implant Therapies in the Oral Cavity Research Group, Complutense University, 28040 Madrid, Spain

**Keywords:** odontogenic sinusitis, SNOT-22, Lund–Mackay scoring, smoking, purulence, maxillary sinus

## Abstract

**Background:** Rhinosinusitis is an increasingly prevalent condition that compromises patients’ quality of life and imposes a substantial burden on healthcare systems. Odontogenic sinusitis, a predominantly unilateral form of rhinosinusitis, accounts for up to 40% of unilateral maxillary sinusitis cases. This study aimed to characterize the clinical presentation, radiological extension, and quality-of-life impairment associated with odontogenic sinusitis. **Methods:** Thirty-eight patients with unilateral sinusitis caused by dental pathology/treatment were recruited. OS diagnosis criteria were established according to EPOS-2020 guidelines. The 22-item SNOT-22 questionnaire was used to assess symptom severity and social/emotional impact. In addition, all patients underwent CT imaging and were staged with the Lund–Mackay scale for involvement of the maxillary, anterior and posterior ethmoid, sphenoid and frontal sinuses. **Results:** Smoking was associated with significantly worse patient-reported quality of life, with higher SNOT-22 scores in current smokers than in non-smokers (59.4 vs. 38.2; *p* = 0.026). Purulent discharge was associated with greater radiological severity, longer symptom duration, and a markedly higher prevalence of extra-maxillary extension (92.3%; *p* < 0.001). Extra-maxillary involvement was identified in 71.1% of patients, predominantly affecting the anterior ethmoid sinus. Although Lund–Mackay scores objectively reflected radiological disease extension, their correlation with symptom burden was limited. **Conclusions:** Odontogenic sinusitis frequently presents with substantial extra-maxillary extension and significant impairment of quality of life. Smoking and purulent discharge were associated with markers of greater disease severity in this exploratory cohort. These associations should be interpreted cautiously and confirmed in larger studies using multivariable analyses.

## 1. Introduction

Rhinosinusitis is an increasingly diagnosed condition impacting 10% to 20% of the population in industrialized countries, significantly compromising their daily quality of life (QoL) and exerting a serious strain on public healthcare systems [1,2].

Quality of life (QoL) represents a multidimensional concept encompassing physical, emotional, social, and functional well-being. Chronic sinonasal conditions may substantially impair daily activities, sleep quality, cognitive performance, emotional status, and social interaction. Similar quality-of-life deterioration has also been described in patients affected by chronic oral and maxillofacial conditions. In odontogenic sinusitis, the coexistence of facial pain, nasal obstruction, purulent discharge, sleep disturbances, and prolonged diagnostic delay may significantly increase disease burden and negatively affect patient-reported outcomes [3,4].

In the latest European position paper on rhinosinusitis and nasal polyps (EPOS 2020), rhinosinusitis was defined as nasal and paranasal sinus inflammation, characterized by two or more symptoms, one being nasal obstruction/congestion/discharge, associated with facial pain/pressure and/or hyposmia/anosmia. Supporting evidence is provided by nasal endoscopy or imaging findings via computed tomography (CT). Rhinosinusitis is considered chronic when symptoms persist for more than 12 weeks [3].

Under the rhinosinusitis spectrum falls odontogenic sinusitis (OS), a predominantly unilateral rhinosinusitis, implicated in up to 75% of unilateral maxillary sinusitis cases. Its identification may prove convoluted due to its heterogeneous etiology and the non-specificity of the elicited symptomatology [5,6]. OS stems from dental/oral pathoses or iatrogenic processes undertaken in the vicinity of the maxillary sinus, triggering mucosal inflammation and hyperplasia, barrier dysfunction, impaired mucociliary clearance, and mucus hypersecretion [7,8]. These processes contribute to tissue fibrosis potentially compromising the patency of the ostio-meatal complex (OMC) causing sinus drainage impairment, and potential spread to other paranasal sinuses [9,10,11,12].

Despite its prevalence (10% to 40% of maxillary rhinosinusitis cases), OS origin is often overlooked in otolaryngology consultations and its complications insufficiently managed in primary dental care [5,13].

Most previous studies on odontogenic sinusitis have focused primarily on etiological factors and treatment strategies, while the relationship between radiological extension, symptom burden, and patient-reported quality of life remains insufficiently characterized. Furthermore, limited prospective data are available regarding the association between clinical features such as smoking status, purulence, and extra-maxillary spread patterns. A more comprehensive understanding of these factors may improve early diagnosis, multidisciplinary management, and therapeutic decision-making in patients with chronic unilateral sinus disease.

This exploratory prospective observational study aimed to evaluate the clinical presentation, radiological extension patterns, and quality-of-life impairment associated with odontogenic sinusitis using the Sino-Nasal Outcome Test questionnaire (SNOT-22) [14] and the Lund–Mackay (LM) staging system [15]. Additionally, the study explored potential associations between symptom burden, radiological severity, smoking status, purulence, and extra-maxillary extension in order to identify clinical features potentially associated with more extensive disease.

## 2. Materials and Methods

### 2.1. Study Design

A group of 38 consecutive patients with a diagnosis of sinusitis originated by dental pathology/treatment was included in this observational prospective study approved by the Research Ethics Committee of San Carlos Hospital-Madrid (22/190-E;13/07/2023), and conforming to the Helsinki Declaration’s ethical provisions. The patients presented to the oral surgery and implantology department clinic at the Madrid Complutense University’s Faculty of Dentistry from July 2023 to June 2024 and provided informed consent.

### 2.2. Size Collection

Given the exploratory nature of this prospective observational study, no formal hypothesis-testing sample size calculation was performed. The study included all consecutive patients who fulfilled the inclusion criteria during the recruitment period. Therefore, the sample should be considered a convenience sample intended to characterize the clinical, radiological, and quality-of-life features of odontogenic sinusitis and to generate hypotheses for future confirmatory studies.

Patient selection was conducted by a multidisciplinary team (a dentist, an oral surgeon, and an ENT specialist) with sinusitis diagnosis based on EPOS-2020 criteria [3]. Patients legally aged with unilateral sinusitis linked to a dental factor were included. Excluded were patients with a history of chronic rhinosinusitis, fungal or allergic rhinosinusitis, previous endoscopic sinus surgery or other sinonasal surgical procedures unrelated to the odontogenic etiology under investigation, sinonasal tumors, or systemic diseases affecting the paranasal sinuses (e.g., cystic fibrosis, primary ciliary dyskinesia, vasculitis or previous radiotherapy); patients who were pregnant; and patients with CT images showing severe artifacts preventing adequate radiological assessment.

The SNOT-22 questionnaire (2006, Washington University, St. Louis) was administered by the examiners through direct interviews with the patients, using a standardized data collection form consisting of 22 items that assessed symptom severity and social/emotional impact over the past two weeks ranging on a Likert-type scale from 0 (“No problem”) to 5 (“As bad as it can be”). The questionnaire was divided into five domains to identify the most affected areas: the nasal domain (items 1–7), ear/facial (8–12), sleep (13–16), concentration/productivity/function (17–19) and psycho-social (20–22). The SNOT-22 score threshold for pathologic values was 9 points [14].

The standard LM staging system [15] widely used in otorhinolaryngology was applied to quantify rhinosinusitis severity via CT scans. Each sinus group (maxillary (Mx), anterior ethmoid (AE), posterior ethmoid (PE), frontal (Frt), and sphenoid (Sph)) is scored from 0 (normal) to 2 (complete opacification), while the OMC is scored 0 (patent) or 2 (obstructed). The total score ranges between 0–12 per side. To minimize errors in the tomographic evaluation, two examiners jointly reviewed the Lund–Mackay classification criteria and conducted a calibration session using 15 CT scans that were not included in the final analysis. Initial discrepancies were discussed until homogeneous criteria for radiological interpretation were achieved. Subsequently, the CT scans included in the study were assessed, and inter-examiner agreement was determined using Cohen’s weighted kappa coefficient for ordinal categories and Cohen’s simple kappa coefficient for the dichotomous variable of extra-maxillary extension. For all estimates, 95% confidence intervals were calculated. Purulence and mucosal edema were evidenced subsequent to endoscopic examination.

Independent variables were gender, age, smoking habits, duration of the symptoms before OS diagnosis and prior treatments. The outcome variables comprised the SNOT-22 scores to assess the degree of impairment caused by OS, and the LM scoring to assess the degree of paranasal sinuses occupancy and the OMC patency. The data obtained are reported in compliance with the STROBE guidelines.

### 2.3. Statistical Analysis

The software IBM-SPSS 24 (IBM, Armonk, NY, USA) was used for statistical processing. The chi-square test and Yates’ correction were employed for categorical variables, while the Kruskal–Wallis test was used to assess the correlation of the means between dependent and independent variables. A correlation analysis was conducted using the Pearson test and Spearman’s Rho correlation. Bonferroni correction in multiple pairwise comparisons to control for type I error inflation was applied. The threshold of statistical significance was set at *p* ≤ 0.05.

## 3. Results

### 3.1. Study Population Characteristics

The study included 38 participants, with a slight male predominance (20 men, 18 women) and a mean age of 49.26 ± 9.94 years (25–79 years). Patients over 45 represented 71.1% of the sample (≤45 years 28.9%). Current smokers represented 44.7% of the participants, 7.9% were former smokers and 47.4% were non-smokers. The affected side was nearly balanced (54% left, 46% right). The symptom duration averaged 23.47 months.

Regarding prior treatments, 45.5% had received antibiotics (ATBs), 39.4% had received dental treatment (DT), 9.1% had received intranasal glucocorticoids (GCCs) and 6% had no prior treatment (NoT). Purulence was observed in 68.4% of cases, while edema was present in 13.1%. The mean SNOT-22 score was 48.71 ± 22.84, and the LM score averaged 6.21 ± 3.13 (Table 1).

The most frequent etiological factors associated with the OS in the sample were periapical cysts, in 26.3% (10 patients) of all cases. Periodontal diseases followed with 23.7% (9 patients), and endo-periodontal lesions were incriminated in 18.4% of cases (7 patients). Post-extraction oroantral communications also represented 18.4% (7 patients) of the etiologies involved, whereas foreign body retention (7.9%; 3 patients) and sinus augmentation procedure (5.3%; 2 patients) were the least implicated in this sample (Figure 1).

### 3.2. SNOT-22 Scores

The mean SNOT-22 of the study population was 48.71 ± 22.84, with no significant gender differences (men: 47.1 ± 25.16, women: 50.5 ± 20.5), though women reported higher, yet non-significant trends in sleep (8.90 against 6.44 in men) and functional domains (7.15 against 5.22 in men) (Table 2).

No correlation was established between SNOT-22 scores and age (rho = –0.206, *p* = 0.215). However, younger patients (<45 years) showed a slightly lower overall symptom burden (44.00 ± 22.79) than those above 45 (51.00 ± 22.84) but significantly more nasal obstruction (rho = –0.525; *p* < 0.001) and facial/otologic symptoms (rho = –0.328; *p* = 0.045).

Symptom distribution analysis revealed the nasal domain as the most affected (22.26 ± 8.92), comprising 45.7% of the total burden, followed by facial/otologic (8.45 ± 4.73) and sleep disturbances (7.74 ± 6.61). Functional (6.24 ± 5.13) and psychological domains (5.79 ± 4.93) showed a lower yet notable impact, but when combined, they yielded a mean score of 12.03 ± 9.57, indicating a substantial effect on QoL.

Gender-based stratification showed higher nasal and facial/ear scores in women than in men (23.72 ± 7.92 vs. 20.95 ± 9.84 and 9.78 ± 4.15 vs. 7.25 ± 5.99, respectively), while men reported higher sleep-related and functional impairment (8.90 ± 6.12 vs. 6.44 ± 6.64 and 7.15 ± 4.78 vs. 5.22 ± 5.06 respectively). Psychological scores were comparable (men: 5.95 ± 5.33; women: 5.61 ± 4.69), though the combined function–psychological domain showed slightly higher impact in men (13.10 ± 10.46 vs. 10.83 ± 8.62). Total SNOT-22 scores were marginally higher in women, though without clear statistical significance (Figure 2).

### 3.3. SNOT-22 and Purulence

Endoscopic examination revealed purulent secretions in 26 patients (68.4%). Symptom severity measured by SNOT-22 was consistently higher in purulent cases across all domains. The mean total SNOT-22 score in purulent cases was 52.85 ± 21.55, compared to 39.75 ± 23.89 in non-purulent cases; patients with purulent discharge “tended to exhibit higher” SNOT-22 scores (mean difference ≈ 13 points), although this difference did not reach statistical significance.

The nasal domain showed the largest score in patients with purulent secretions (24.19 ± 7.92 against 18.08 ± 9.84 without purulence), followed by the sleep domain (8.85 ± 6.64 against 5.33 ± 6.12 without purulence).

Functional impairment in purulent cases scored higher (7.23 ± 4.52) than in non-purulent cases (4.08 ± 5.58) without reaching statistical significance. However, within this domain, items like concentration or nasal congestion/obstruction registered significantly higher scores in the presence of purulence (2.27 ± 1.66 and 2.6 ± 4.42) than in its absence (1 ± 1.54 and 1.2 ± 3.08) (*p* = 0.023 and *p* = 0.008, respectively).

The psychological domain demonstrated a similar trend, with higher values in purulent cases (6.46 ± 4.69 vs. 4.33 ± 5.33, *p* = 0.164). This pattern persisted when combining functional and psychological domains (13.69 ± 9.18 vs. 8.42 ± 9.79). The facial/ear domain showed the smallest difference between groups (8.62 ± 4.15 vs. 8.08 ± 5.99), suggesting minimal influence of purulence on this symptom cluster.

### 3.4. LM Scores

The obtained LM scores were supported by the kappa coefficient analysis, which showed a weighted kappa value of 0.81 for the ordinal scores and a kappa value of 0.86 for the extra-maxillary localization.

In our cohort of 38 patients, the mean total LM score was balanced through genders: 6.25 ± 3.06 in men vs. 6.17 ± 3.29 in women (Figure 3).

Despite minor variations in subregions (LM right-Mx, LM right-AE, LM right/left-OMC), while total LM scores did not correlate with age (≤45 years: 6.64 ± 2.77; >45 years: 6.33 ± 3.13), older patients exhibited greater opacification in specific regions. Those with total opacification of right-Frt had a mean age of 60.2 ± 11.1 years (47.53 ± 8.93 without opacification, *p* = 0.039), while patients with right-PE opacification averaged 68.5 ± 14.85 years (48.57 ± 8.06 without opacification, *p* = 0.018). Similarly, age distribution differed between maxillary and extra-maxillary involvement. Patients with extra-maxillary involvement at the right nasal fossa were older than those who showed only maxillary involvement (*p* = 0.011). Laterality analysis showed a slightly higher mean age in extra-maxillary cases on the right side (*n* = 13; 51.08 ± 12.68 years) than maxillary cases (*n* = 25; 48.32 ± 8.33 years,), though these means are close to the mean age of the sample.

### 3.5. LM Scores, Purulence and Extra-Maxillary Extension

Purulent cases had significantly higher total LM scores (7.77 ± 2.27 against 2.83 ± 1.75 in non-purulent cases, *p* < 0.001), indicating greater sinus opacification (Table 3, Figure 2). On the left side, opacification was notably higher in purulent cases (4.19 ± 4.35 vs. 0.75 ± 1.42), reflecting its radiologic impact. The AE was most affected after the Mx sinus, with 79.5% showing partial or total opacification (Table 3). Purulence was strongly associated with extra-maxillary extension, which was present in 92.3% of purulent cases vs. 25% of non-purulent cases (chi-square = 18.085, *p* < 0.001).

OMC obstruction was present in 84.6% of cases, with 46.1% on the right and 38.5% on the left, and 15.4% unaffected. Among purulent cases, 96.3% had OMC obstruction against 58.3% in non-purulent cases.

Unexpectedly, patients with edema had lower LM scores (3.20 ± 2.05) than patients clear of edema (6.67 ± 3.03, *p* = 0.019), suggesting edema correlates with symptoms but not increased sinus opacification.

### 3.6. Smoking Status

Current smokers reported the highest average SNOT-22 scores (59.44 ± 18.29), while non-smokers displayed the lowest scores (38.22 ± 22.29), with former smokers falling in between (43 ± 19.16) (*p* = 0.026) (Figure 4).

Furthermore, pairwise comparison indicated an important statistically significant difference between current and never smokers (*p* = 0.009). Smokers registered higher scores, particularly in the nasal domains of SNOT-22, with a mean of 21.88 ± 7.13 and 11.29 ± 3.98 in the facial/ear domain, compared to non-smokers (16.83 ± 8.63 in the nasal domain, and 6.94 ± 4.23 in the facial/ear domain), without these values reaching statistical significance.

Smokers showed a higher mean total LM-score (7 ± 3.14; non-smokers: 5.61 ± 3.09) and opacification in specific regions, particularly the Mx and AE/PE sinuses. However, no specificities were reported for smokers in terms of purulence, edema or symptom duration (Table 4).

### 3.7. Previous Treatments and Symptoms Duration

The mean symptom duration reported in this study (23.47 months) reflects a chronic disease course. A share of 94% of the patients received previous treatments including ATBs, nasal GCCs or DT. Patients treated with nasal GCC experienced longer symptom durations (30.67 ± 26.63 months) compared to NoT patients (7 ± 7.07 months). The ATB group showed a high mean symptom duration (26.61 ± 16.79), but this was lower than for the GCC group, whereas the DT group showed a shorter symptom duration (19.56 ± 19.80) than both. Patients with purulence had prolonged symptoms (26.88 ± 9.66 months) compared to those with normal endoscopic findings (16.08 ± 14.6 months) (Figure 5).

Symptom duration was generally longer in patients with partial or total sinus opacification than in those without sinus opacification, though the overall differences regarding symptoms’ duration were not statistically significant.

## 4. Discussion

OS remains underdiagnosed and is often mismanaged, leading to prolonged disease course and ineffective treatment pathways, entailing profound implications for patients’ QoL. The SNOT-22 questionnaire is widely employed to assess CRS symptom burden but is scarcely used for sinusitis of various dental origins. In this study it was clustered into five domains, and LM scoring was segmented to assess extra-maxillary extension, enabling a precise radiographic evaluation of paranasal sinus involvement and a thorough assessment of the condition’s clinical implications within the study population.

Although inflammatory periapical and periodontal diseases, oroantral communications, sinus augmentation procedures, and displaced dental materials remain the most common causes of odontogenic sinusitis, less frequent etiologies should also be considered. Medication-related osteonecrosis of the jaw (MRONJ), particularly involving the maxilla, may lead to oroantral communication, chronic bone exposure, and secondary maxillary sinusitis. Although uncommon, this condition should be included in the differential diagnosis of unilateral chronic maxillary sinusitis, especially in patients receiving antiresorptive or antiangiogenic medications [16,17].

### 4.1. SNOT-22 Domains and LM Scores

The highest SNOT-22 scores recorded were in the nasal domain, and the lowest in the psychological (emotional) domain. This aligns with Kwiatkowska et al. [18], where the highest and lowest SNOT-22 scores correspond to the same domains of the present study, although their research focused only on OS caused by periapical lesions. QoL-related domains (sleep, function and psychological) when combined yielded a score of 19.45 ± 14.87, underscoring a serious disease burden, as concluded in the studies of Gaudin et al. [19] and Kwiatkowska et al. [18]. Simuntis et al. [20] reported even higher emotional distress in OS than in rhinogenic sinusitis.

In the present study, the extra-maxillary extension registered was substantial, with AE involvement reaching 79.5% and Frt sinusitis in almost 41%, likely due to the prolonged symptom duration in the study cohort (23.47 months), promoting disease progression. Fadda et al. [21] reported lower AE involvement (51.6%, and 12.9% of the Frt sinus) while Saibene et al. [22] found 41% experienced unilateral extra-maxillary extension. Goyal et al. [23] identified Frt sinus involvement as a diagnostic indicator of OS over CRS.

While LM scoring objectively complemented SNOT-22 assessment, the correlation between both metrics was inconclusive within this sample. This finding is consistent with previous chronic rhinosinusitis literature, suggesting that radiological severity does not necessarily correlate with subjective symptom burden or quality-of-life impairment. Therefore, radiological findings should be interpreted together with clinical symptoms and patient-reported outcomes rather than as isolated indicators of disease severity.

Although conventional computed tomography remains the gold standard for comprehensive evaluation of odontogenic sinusitis due to its superior assessment of sinonasal anatomy and extra-maxillary extension, cone-beam computed tomography (CBCT) has gained increasing relevance in dental and maxillofacial practice because of its lower radiation dose, shorter acquisition time, lower cost, and high spatial resolution for osseous structures. CBCT has demonstrated reliability for the anatomical evaluation of maxillofacial and sinonasal structures and may represent a valuable imaging alternative for future odontogenic sinusitis studies performed in dental settings. Nevertheless, its limitations regarding soft-tissue contrast compared with conventional CT should be acknowledged [24].

### 4.2. Purulence

Multiple studies have identified purulence in the middle meatus as a hallmark of OS, particularly in acute cases, where it appears in up to 58% of patients [25,26,27]. In the present study, 68.4% of the patients presented purulence at the endoscopic observation, a very similar rate to that found by of Costa et al. [28] (two-thirds of the study population had purulence) and Aljeraisi [29] (70%).

Total SNOT-22 scores were 13.1 points higher in patients with purulence, reflecting a severe impact on nasal function and QoL (sleep quality, performance status). This finding may inform clinical evaluation and therapeutic strategy development for OS patients.

Previous studies on chronic rhinosinusitis have consistently demonstrated that the presence of purulent nasal discharge is associated with greater symptom burden, poorer disease-specific quality of life, and increased disease severity. Our findings suggest that a similar pattern is also observed in odontogenic sinusitis, as patients presenting purulent discharge exhibited higher SNOT-22 scores across all evaluated domains. However, unlike non-odontogenic chronic rhinosinusitis, odontogenic sinusitis is sustained by a persistent dental infectious focus, typically unilateral, which may promote ongoing inflammation, obstruction of the osteomeatal complex, and a greater propensity for extra-maxillary extension. These pathophysiological differences may explain the high prevalence of extra-maxillary involvement observed in our cohort, despite the limited correlation between radiological severity and patient-reported symptom burden [3,19,20,30].

Robinson et al. [31], in their study, found an absence of correlation between purulence and LM scores, contrasting with our findings, where elevated LM scores were registered in the presence of purulence.

Purulence also correlated with extensive sinus involvement patterns, which were present in 92.3% of purulent cases (*p* < 0.001). Similarly, longer symptom duration was observed in patients presenting purulence, likely due to a pathophysiological cascade of persistent inflammation leading to suppuration; in turn, this impairs sinus outflow and promotes both persistence and trans-sinusal progression of the disease, reinforcing purulence as a marker of advanced OS and more extensive sinus involvement.

OMC obstruction is a key criterion in endoscopic sinus surgery decision-making [32]. Sabatino et al. [33] reported it in 42% of cases, while in our study, 84.6% of the study participants had OMC obstruction, and this was also observed in 96.3% of patients with evidenced purulence. These findings highlight the need to assess OMC patency when considering surgical intervention in OS cases.

### 4.3. Smoking Status

To date, various studies have addressed smoking status as a covariate contrastingly impacting surgical outcomes in CRS [30,34]. Gill et al. [35] observed that tobacco increases the CRS risk 1.42- to 3.60-fold while Tan et al. [36], in a meta-analysis including 45 studies, found that smokers and former smokers had poorer baseline QoL compared to never-smokers.

This study explored the association between smoking status and symptom burden, quality of life, and radiological severity in odontogenic sinusitis. Current smokers presented significantly higher SNOT-22 scores than never-smokers and showed a trend toward higher Lund–Mackay scores. However, these findings derive from unadjusted bivariate analyses and may therefore be influenced by residual confounding factors, including age, symptom duration, purulence, previous treatment, and disease extension. Furthermore, in terms of smoking exposure, study participants were classified only as current, former, or never smokers, without quantitative measurement of pack-years or smoking cessation time. Consequently, these findings should be interpreted as exploratory and hypothesis-generating, and require confirmation in larger prospective studies incorporating multivariable analyses.

### 4.4. Previous Treatments and Symptom Duration

Despite 94% of the patients receiving previous treatments (ATBs, DT or GCCs), symptoms persisted, highlighting limited effectiveness when underlying dental causes remain unaddressed. The patients receiving either nasal GCCs or ATBs showed the longest symptom duration. In OS, this aligns with expert consensus that ATBs alone are inadequate to reach a conclusive recovery in OS, and that non-surgical treatments should be reserved for acute-phase management while dental treatments are conducted [10,37].

In OS, elimination of the dental focus is the first-line treatment. Endoscopic sinus surgery (ESS) is indicated when symptoms or obstruction persist after dental therapy, or in the presence of foreign material, oroantral communication, or extra-maxillary involvement (ethmoid or frontal sinuses). In limited maxillary OS, a wide middle meatal antrostomy usually provides adequate ventilation and drainage. If ethmoidal or frontal recess disease is present, an extended ESS (e.g., anterior ethmoidectomy or complete ESS) may be necessary, tailored to the anatomical and pathological extent [10,13].

In contrast, patients who received DT reported shorter symptom durations, likely due to the immediate referral to an ENT (ear, throat, nose) specialist in instances of symptom persistence after dental cause elimination. Intriguingly, patients without prior treatment exhibited the shortest symptom duration, possibly due to a milder disease form or timely diagnosis.

Finally, several limitations of this study should be acknowledged. First, the cohort mainly included patients with prolonged symptom duration and chronic disease presentation, which may not fully represent early-stage odontogenic sinusitis. Second, although the sample size was adequate for the primary exploratory analyses, the relatively limited number of patients may have reduced the statistical power of subgroup comparisons. Furthermore, the analyses were primarily based on unadjusted bivariate comparisons. Consequently, the observed associations between smoking status, purulence, symptom burden, radiological severity, and extra-maxillary extension may have been influenced by residual confounding. Larger prospective studies with sufficient statistical power should incorporate multivariable analyses to determine the independent contribution of these clinical variables. Third, endoscopic evaluation was performed within the routine clinical diagnostic setting and not under blinded conditions, which may represent a potential source of observational bias. In addition, anatomical variables potentially influencing sinonasal ventilation, such as nasal septal deviation, were not systematically assessed.

In addition, in terms of smoking exposure, study participants were categorized only as current, former, or never smokers. Quantitative measures of tobacco exposure, such as pack-years and time since smoking cessation among former smokers, were not systematically recorded. Consequently, the observed associations between smoking status and symptom burden or radiological severity should be interpreted with caution. Future studies should incorporate a more detailed assessment of smoking exposure to evaluate potential dose–response relationships and better define the role of tobacco use in odontogenic sinusitis.

Despite these limitations, the present findings suggest that a structured evaluation of symptom burden and radiological extension may contribute to a more comprehensive clinical characterization of odontogenic sinusitis, facilitating the identification of patients presenting more extensive disease and potential extra-maxillary involvement at the time of diagnosis. Given the observational nature of the present study, these findings should be considered hypothesis-generating and require confirmation in larger prospective multicenter cohorts with longitudinal follow-up.

The broad inclusion of odontogenic etiologies additionally highlights the need for future studies stratified according to specific dental pathologies and procedures, which may reveal more detailed patterns of disease severity, radiological spread, and quality-of-life impairment. Future investigations should also incorporate additional clinical and radiological parameters, including the characteristics and extent of odontogenic lesions, microbiological findings, anatomical factors such as sinonasal anatomical variations, treatment modalities, and patient-related variables that may influence disease presentation, progression, and treatment outcomes. Larger prospective multicenter studies integrating these variables could contribute to the development of more comprehensive diagnostic and prognostic models for odontogenic sinusitis [38].

Such studies may ultimately contribute to the development of evidence-based risk stratification strategies and more individualized multidisciplinary management of patients with odontogenic sinusitis.

## 5. Conclusions

Odontogenic sinusitis is a complex condition that requires close collaboration between dental and otolaryngology specialists. Combined assessment using patient-reported outcomes (SNOT-22) and radiological staging (Lund–Mackay) provides complementary information for evaluating disease burden and extension. Although radiological severity was not directly associated with symptom burden, smokers and patients presenting purulent discharge exhibited greater quality-of-life impairment and more extensive extra-maxillary disease. Early identification of these potentially high-risk clinical features may facilitate multidisciplinary clinical assessment and treatment planning. Future prospective studies including therapeutic follow-up are warranted to determine whether these factors influence clinical outcomes and disease progression.

## Figures and Tables

**Figure 1:**
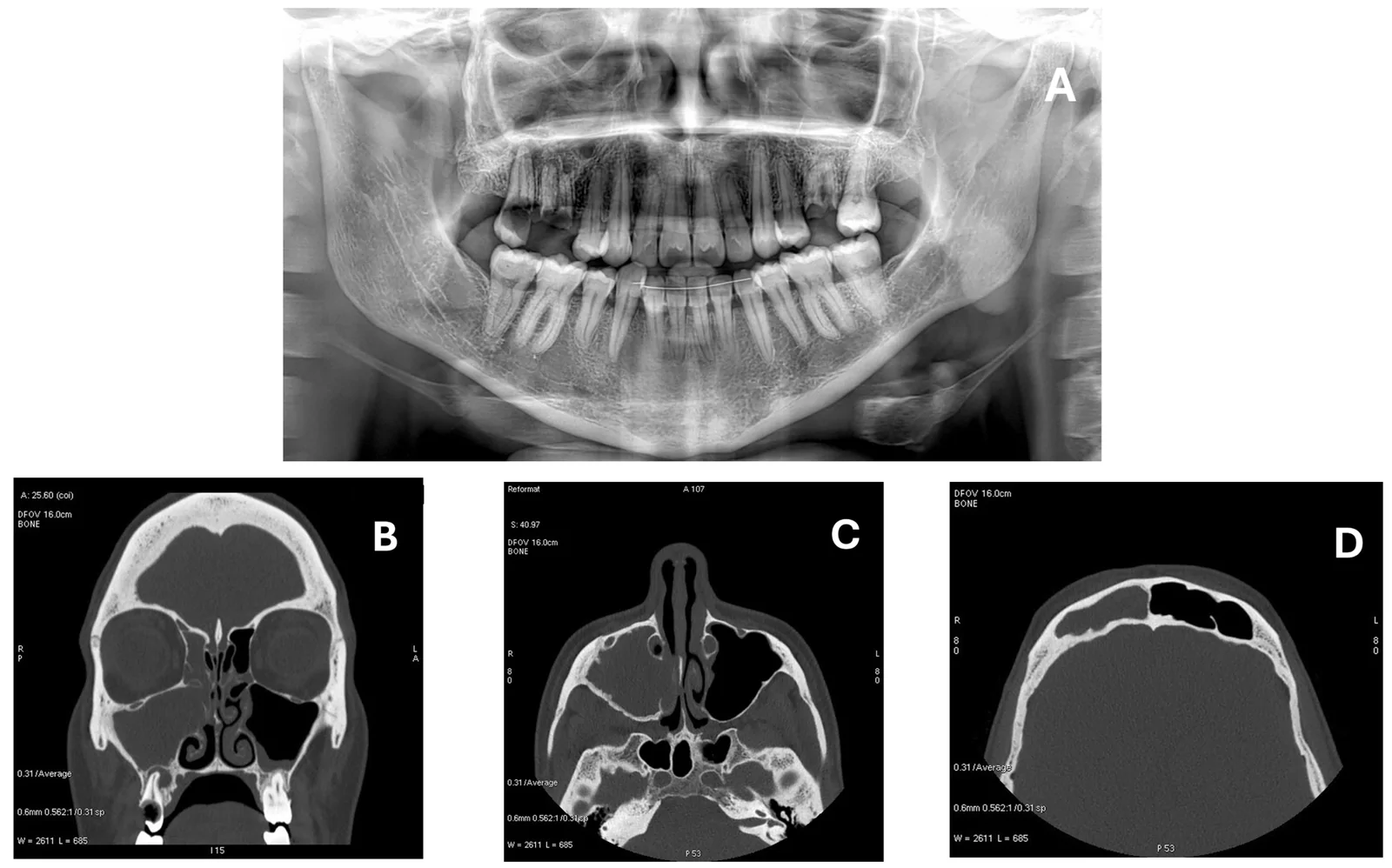
Patient with lesions involving the right maxillary molar and premolar, identifiable on the panoramic radiograph (**A**). Coronal and axial CT images demonstrate involvement of the maxillary sinus and extra-maxillary extension (**B**–**D**).

**Figure 2:**
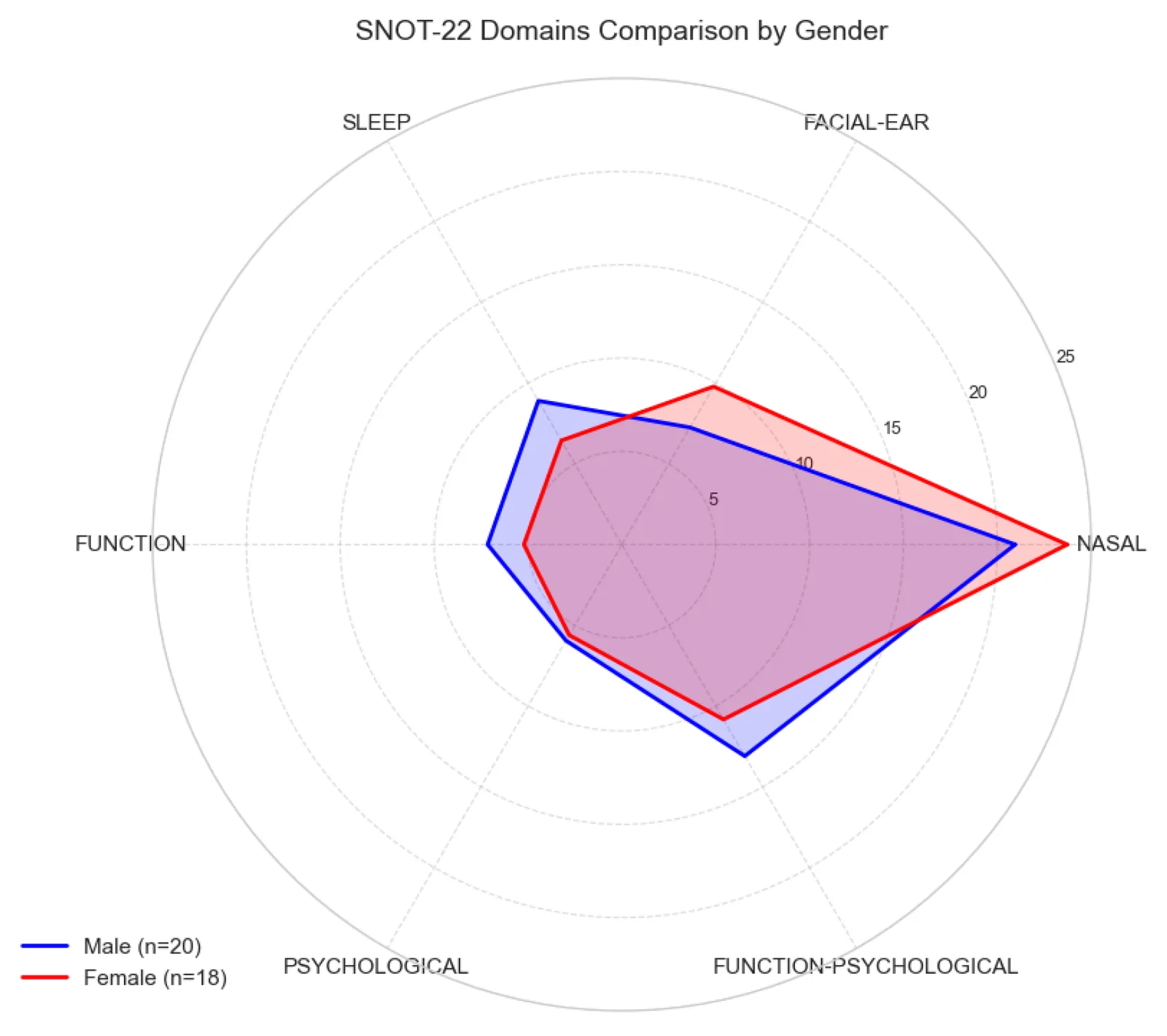
Radar plot representing the distribution of SNOT-22 domain scores across genders.

**Figure 3:**
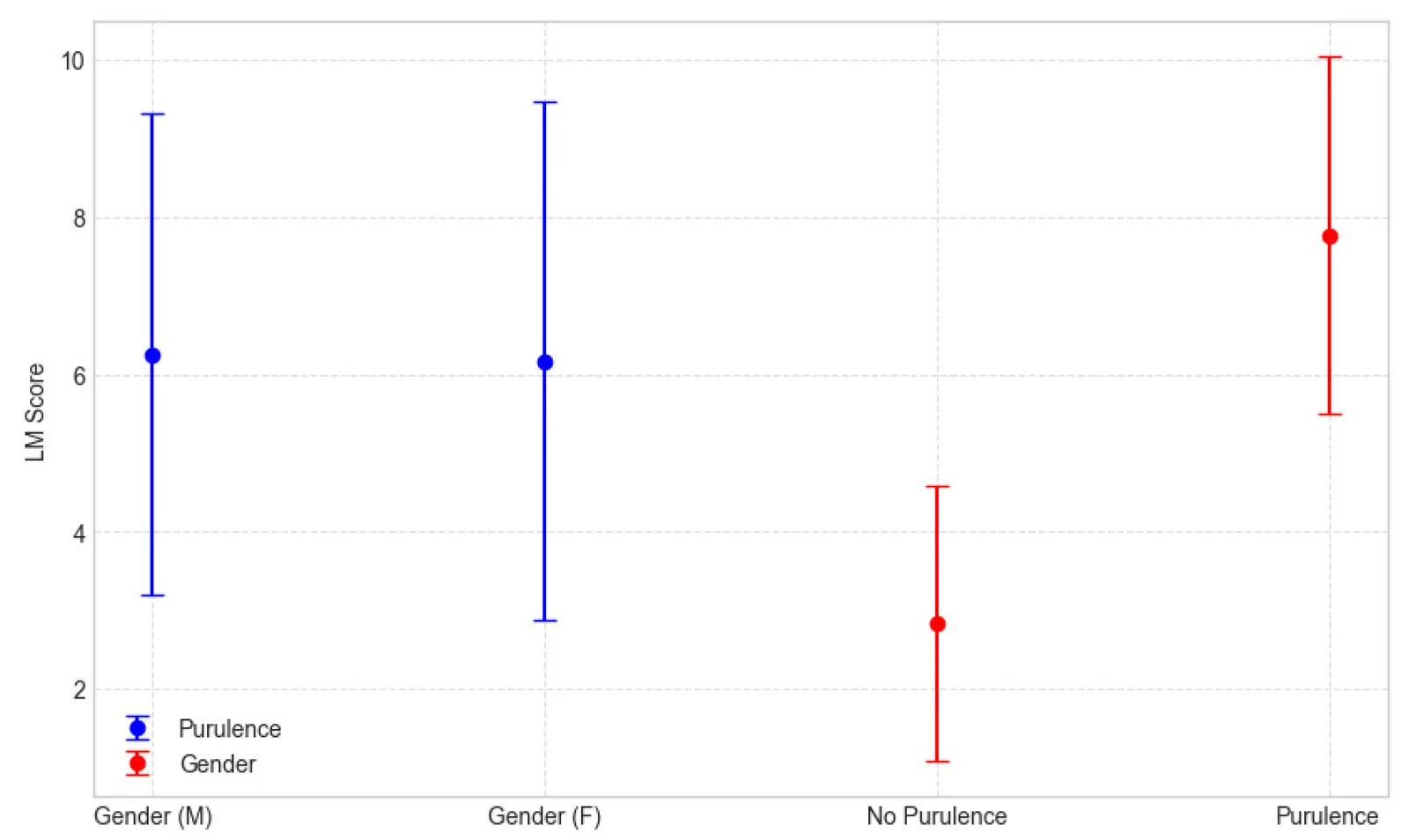
LM score distribution as a function of gender and presence of purulence.

**Figure 4:**
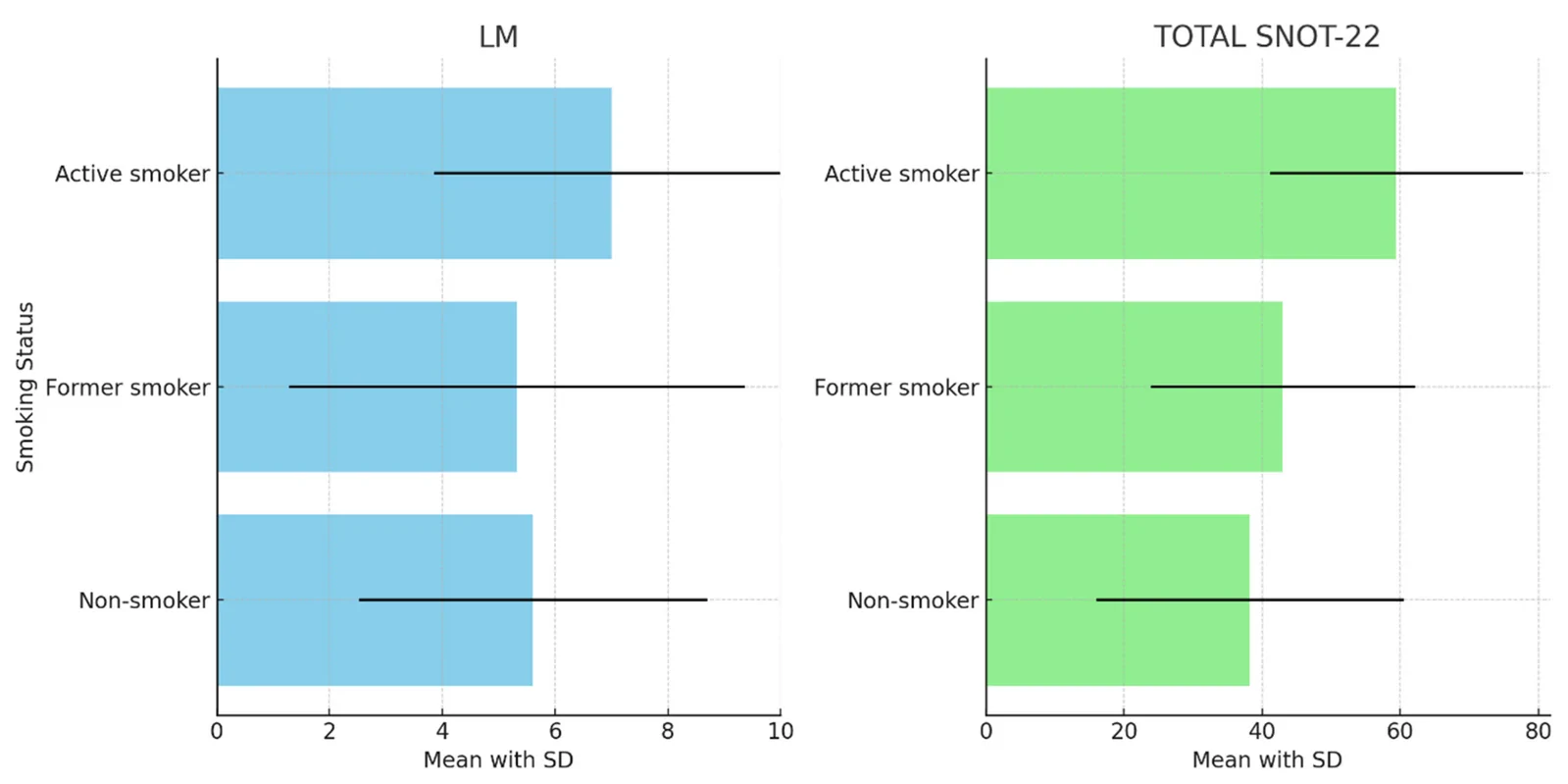
Bar chart of mean LM and SNOT-22 score with standard deviation across smoking status groups.

**Figure 5:**
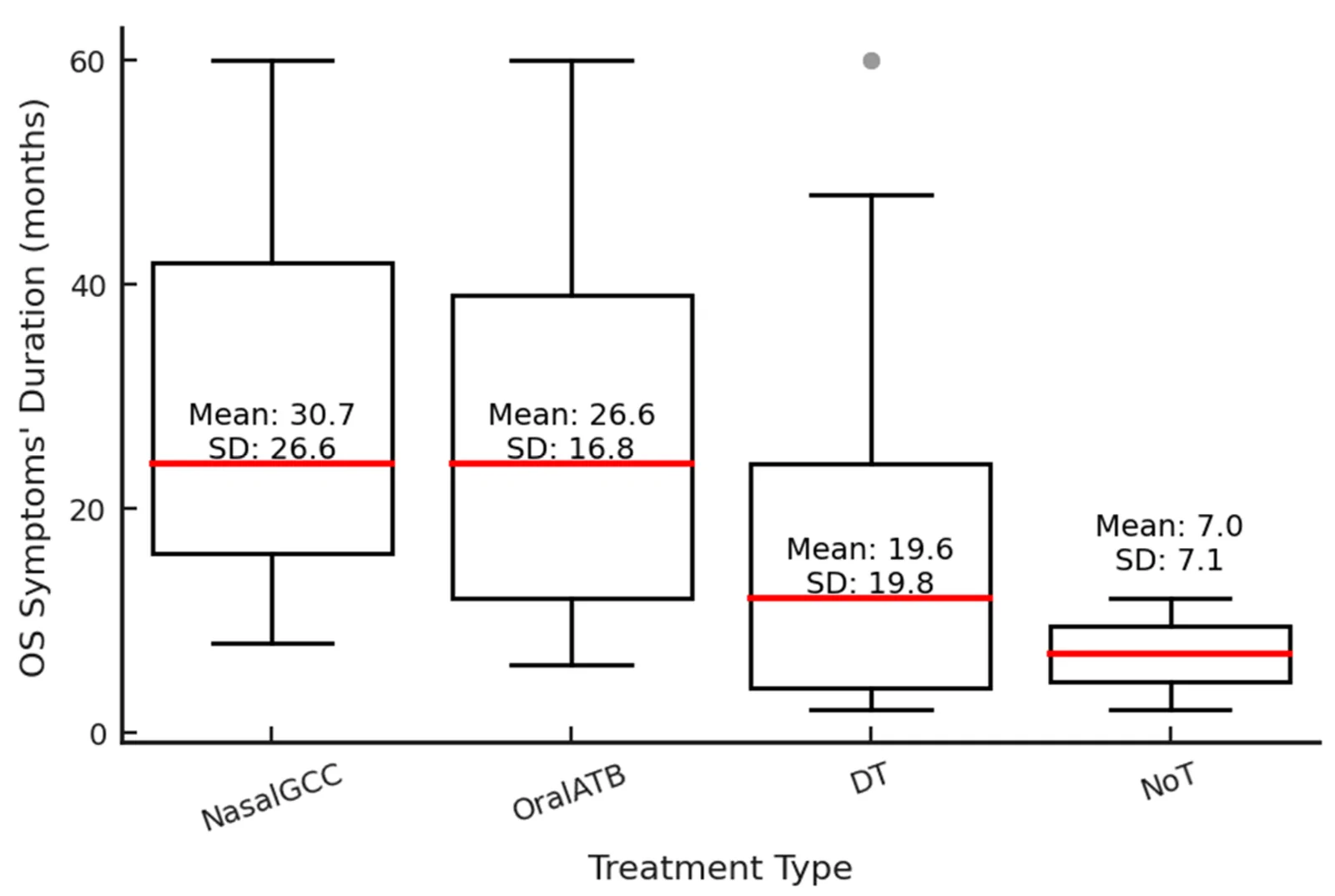
Boxplot representing the duration of symptoms in groups of patients according to the previous treatments they received. SD: standard deviation, Nasal GCC: nasal glucocorticoid, Oral ATB: oral antibiotic, DT: dental treatment, NoT: no treatment.

**Table 1:** Characteristics of the study population.

Gender	M: 52.6% (20 patients) F: 47.4% (18 patients)
Age	49.26 ± 9.94; [25–79 years]
Smoking Habits	NS: 47.4% (18 patients) FS: 7.9% (3 patients) CS: 44.7% (17 patients)
Affected Side	L: 54% (21 patients) R: 46% (17 patients)
Symptom Duration	23.47 ± 18.73 months
Previous Treatments	ATB: 45.5% (17 patients) DT: 39.4% (15 patients) GCC: 9.1% (4 patients) NoT: 6% (2 patients)
Purulence	Yes: 68.4% (26 patients) No: 31.6% (12 patients)
Edema	Yes: 13.1% (5 patients) No: 86.9% (33 patients)
Mean SNOT-22	48.71 ± 22.84
Mean Lund–Mackay	6.21 ± 3.13

Abbreviations: M: male, F: female, NS: non-smoker, FS: former smoker, CS: current smoker, L: left, R: right, ATB: antibiotic, DT: dental treatment, GCC: glucocorticoid, NoT: no treatment.

**Table 2:** SNOT-22 domain mean scores and standard deviations stratified by gender and purulence at endoscopic examination.

SNOT-22 Domain	Gender	N	Mean	SD	*p*	Purulence	N	Mean	SD	*p*
Nasal	M	20	20.95	9.84	0.303	NO	12	18.08	9.03	0.071
	F	18	23.72	7.92		YES	26	24.19	8.80	
	Total	38	22.26	8.92		Total	38	22.26	8.92	
Facial/Ear	M	20	7.25	5.99	0.119	NO	12	8.08	3.82	0.327
	F	18	9.78	4.15		YES	26	8.62	5.36	
	Total	38	8.45	4.73		Total	38	8.45	4.73	
Sleep	M	20	8.90	6.12	0.361	NO	12	5.33	7.38	0.106
	F	18	6.44	6.64		YES	26	8.85	5.55	
	Total	38	7.74	6.61		Total	38	7.74	6.61	
Function	M	20	7.15	4.78	0.264	NO	12	4.08	5.58	0.066
	F	18	5.22	5.06		YES	26	7.23	4.52	
	Total	38	6.24	5.13		Total	38	6.24	5.13	
Psychological	M	20	5.95	5.33	0.806	NO	12	4.33	5.03	0.164
	F	18	5.61	4.69		YES	26	6.46	4.96	
	Total	38	5.79	4.93		Total	38	5.79	4.93	
Function-Psychological	M	20	13.10	9.79	0.478	NO	12	8.42	10.46	0.076
	F	18	10.83	9.18		YES	26	13.69	8.62	
	Total	38	12.03	9.57		Total	38	12.03	9.57	
Total SNOT-22	M	20	47.10	23.89	0.718	NO	12	39.75	25.16	0.121
	F	18	50.50	21.5		YES	26	52.85	20.51	
	Total	38	48.71	22.84		Total	38	48.71	22.84	

Abbreviations: SD: standard deviations. M: male, F: female, N: number of patients.

**Table 3:** LM score distribution by gender and purulence, and rates of cases presenting other paranasal sinus involvement.

Gender		N	Mean	SD	*p*	Purulence	N	Mean	SD	*p*	Ex-Mx-Ext	Rate (%)
LM RNF	M	20	4.10	3.70	0.126	No	12	2.08	2.11	0.429	AE	79.5
	F	18	2.00	3.03		Yes	26	3.58	3.95			
	Total	38	3.11	3.52		Total	38	3.11	3.52		PE	46.15
LM LNF	M	20	2.15	3.70	0.2	No	12	0.75	1.42	0.114		
	F	18	4.17	4.16		Yes	26	4.19	4.35		Frt	
	Total	38	3.11	4.01		Total	38	3.11	4.01			41.03
LM Total	M	20	6.25	3.06	0.96	No	12	2.83	1.75	<0.001		
	F	18	6.17	3.29		Yes	26	7.77	2.27		Sph	10.25
	Total	38	6.21	3.13		Total	38	6.21	3.13			

RNF: right nasal fossa, LNF: left nasal fossa, M: male, F: female, N: number of patients, SD: standard deviation, Ex-Mx-Ext: extra-maxillary extension, AE: anterior ethmoid sinus, PE: posterior ethmoid, Frt: frontal sinus, Sph: sphenoid.

**Table 4:** Total SNOT-22 and LM scores in patients according to their smoking status.

Smoking Status	Patients (N = 38)	LM	*p*	SNOT-22	*p*
Non-smokers	*n* = 18 (47.4%)	5.61 ± 3.09	0.363	38.22 ± 22.29	0.026
Former smokers	*n* = 3 (7.9%)	5.33 ± 4.04		43 ± 19.16	
Current smokers	*n* = 17 (44.7%)	7 ± 3.14		59.44 ± 18.29	

## Data Availability

The data presented in this study are available on reasonable request from the corresponding author. The data are not publicly available due to privacy and ethical restrictions.
